# “I Desire to Suffer, Lord, because Thou didst Suffer”: Teresa of Avila on Suffering

**DOI:** 10.1111/hypa.12488

**Published:** 2019-07-09

**Authors:** Noelia Bueno‐Gómez

## Abstract

Teresa of Avila's desire for suffering cannot be interpreted as the mere passive assumption of a feminine sacrificial role. On the contrary, Teresa was able to transform her suffering into the incarnated performance of her relationship with God: By desiring suffering and by understanding it and her ability to confront it as proof of divine love, she was able to reinforce her self‐confidence and strength. This article discusses Teresa of Avila's experience and interpretation of suffering in the context of the female ascetic‐mystic Christian tradition. It criticizes Teresa's positive conceptualization of suffering but examines in depth the potential of her ability to actively manage and control it. Although Teresa was able to affirm her personality through ascetic practices such as self‐humiliation and mortification, the general applicability of such practices to the management of suffering is fraught since they leave the suffering individual in a vulnerable position. Although Teresa of Avila finds fulfillment and, paradoxically, self‐actualization through self‐denial and the surrender of her will, such practices entail the substantial risk of total self‐annihilation.

## Introduction

Teresa of Avila's desire for suffering may seem shocking to our modern sensibilities, shaped as they are by the techno‐scientific era and its dedication to reducing pain through anesthesia and painkillers, fighting any form of illness, improving living conditions, designing better resources for pleasure and joy, and even the complete eradication of suffering.^1^ However, Teresa's work is still a valuable resource for understanding the experience of human suffering under the influence of Christianity during the ensuing centuries, particularly for understanding the experience of women because of the influence of Teresa's writings on female education. After all, as a Carmelite reformer, saint, and one of first two women to achieve the distinction of doctor of the Catholic Church (along with Catalina of Siena), Teresa contributed to the creation of an influential ideal of femininity both in Spain and abroad.

The European ethos is still very much influenced by the Christian tradition, and even though it may not seem so at first glance, this applies to the experience of suffering too. Cultural patterns originally associated with the Christian religious experience of suffering in all its dimensions persist—probably more in the form of assumed beliefs (not always recognizable, even for ourselves),^2^ attitudes, and behaviors than in rituals and official membership in religious institutions (although striking rituals persist, for example, the *flagelantes* studied by Patrik Vandermeersch in the Spanish town of San Vicente de la Sonsierra, La Rioja [Vandermeersch 2014]).^3^ Widespread attitudes like feeling less guilty after “paying” with suffering for a previous infraction,^4^ or the belief that we can better help others if this help entails a certain suffering or inconvenience for us are not at all universal, much less a part of “human nature.”^5^ Although the association of suffering with morality far precedes the Christian tradition (for example, the English word “pain” and the German “Pein” come from the ancient Greek “poine” and the Latin “poena,” meaning “punishment” [Le Breton 1999]; “pena” in Spanish is not only a “[legal] punishment” but also means “sorrow”), Christian tradition has shaped and changed this association.

This article is dedicated to understanding why Teresa of Avila, and the ascetic‐mystic ideal in general, exalts suffering and even seeks it out, and what the feminine particularities of this ideal and its exaltation are. I will explore the “active” character of Teresa's management of suffering, that is, its proactive management and utilization for further purposes. Finally, I will discuss whether there is any advantage or strength to this form of management that may have practical value for postmetaphysical practical philosophy.

## Christian Mysticism and Asceticism on Suffering

In the Christian context, a “mystical experience” can be defined as a personal religious experience in which divinity is felt to be intensely close or joined to oneself, for example, in the form of rapture, ecstasy, or visions. Such experiences can occasionally present as externally visible, physical phenomena (for example, rigidity of the body, elevations, stigmata), but are more commonly felt exclusively by the mystic person, who experiences a kind of liberation and other intense emotions, an increase in his or her faith in God, a particular sudden understanding of religious mysteries (like the Mystery of the Trinity), an ability to foresee the future and/or to become a mediator between God and other human beings. These mystical experiences usually occur in believers living religious lives according to the moral precepts of Christianity, ascetic and partially or totally contemplative, although there is a tradition of ascetic‐mystic women who were not integrated into religious institutions.^6^ God is directly “felt” by the mystic persons rather than “known” rationally, as is the case in intellectual theology (scholasticism).^7^

Women have played an important role in Christianity since its earliest days: Through a woman, the Son of God was born into the world; furthermore, Jesus did not reject the company of women, and it was a group of women who first discovered that his body had vanished from the grave three days after his death, all according to the New Testament. The most loyal interpretations and revitalizations of the original teachings of Jesus (represented by figures like Priscillian, unjustly condemned and accused of heresy [Nuñez García 2016], or Francis and Clara of Assisi) recognized this early significance of the role of women, which was distinctly not incorporated in the institutionalized Catholic Church, where women were soon excluded from the administration of the sacraments, the ecclesiastical authority, and the ministry. Similarly, they were excluded from the debates of intellectual theology, which left women who were looking to deepen their spirituality little recourse beyond looking for direct religious experiences, and they took the way opened by mystic theology. In this context, the idea of Incarnation and the devotion to the humanity of Christ are especially relevant, particularly from the twelfth century onward (Vandermeersch 2014, 71).

Christian religious authorities have always regarded mysticism with a fair bit of suspicion, as the Church has carefully protected its dogma, the revealed truth, and its historical dimension against the possibility of new prophets pretending to possess new authority. Mystic persons have frequently been met with distrust and have even been investigated as possible heretics, particularly by the Catholic Church after the Reformation in the sixteenth century, when independent religious experiences were regarded as suspect for their dismissal of the sacraments and the mediation of the Church between believers and God. Such was the case of Teresa of Avila (Javierre 1982).^8^ Since women's mystic experiences were a particular object of suspicion for ecclesiastical authorities, as in the case of Angela of Foligno or Dorotea of Montau (Gajano 2006), they used extreme mortification to demonstrate the authenticity of their experiences (McGlynn 2011), among other resources.

Suffering is not particularly relevant in mystic experiences. Being so close to God is described as intense and usually full of contradictory emotions; in this sense, it can be a kind of disturbance—for example, Teresa of Avila describes the transverberation as a kind of “soft pain” (V 29, 13).^9^ However, this is not always the case; sometimes the mystical union is simply a joyful state of grace without any lingering discomfort. In fact, a kind of spiritual jubilation, even physical pleasure or the cessation of the senses (temporary inability to see, hear, and so on) are also characteristic of mystical unions. Regardless, suffering plays a much more relevant role in ascetic practices, which are meant to put the individual into the best disposition to achieve divine grace. Interestingly, a majority of mystic persons also underwent intense unintentional suffering in their lives, like painful illnesses or disorders (for example, Hildegard of Bingen) (Carpinello 2006, 68).

The authentic mystic union is considered a gift from God, so it is impossible for human beings to achieve it on their own. But they can prepare themselves, so to speak, for the possibility by demonstrating their readiness for this gift through perfecting their virtuousness. Asceticism is the way of life for individuals looking to perfect the Christian virtues and purify their souls in order to be close to the divinity, particularly by renouncing worldly pleasures and commodities, and—in certain extreme cases throughout history—even to the extent of mutilating their own bodies and impairing themselves.^10^ Asceticism entails self‐improvement: a deep knowledge of oneself, a particular methodical modulation of the self (of one's own thoughts, conscious and unconscious dimensions, deeds, intentions, expectations, and so on) with the intention of better loving God and attaining the divine grace, his love. Salvation can be expected by common believers, but mystical union is reserved for perfect souls.

The general elements of Christian asceticism are the surrender of oneself, the acceptance of the Cross (Luke 9:23), the renunciation of material possessions (Matthew 19:21), the renunciation of all attachments to the world and one's own life (Luke 14:26), and the assumption of the fact that this world is not a home for human beings (Luke 9:58).^11^ When Christians ceased to be prosecuted in the Roman Empire in the fourth century and the martyrdom stopped, a different kind of suffering, derived from asceticism and mortification, was associated with extreme religious practices, as in the case of the Desert Fathers (including Antonio Abad, who founded anchoretic monasticism, and Panchomio, who founded coenobitic monasticism (Goehring 1999)), and also the Desert Mothers or the case of the Stylites, particularly Simenon and Daniel, who lived in Syria in the fourth and fifth centuries, and who practiced extreme mortifications like fasting, long vigils, painful bindings, and standing for long periods.^12^

The tendency toward exacerbation of the ascetic practices in the Christian tradition increased from the twelfth century onward, with a boom during the fifteenth and sixteenth centuries, particularly in the Catholic context. The biographies of Bernard of Clairvaux, Henry Suso, Cathalina of Siena, and Dorothea of Montau describe extreme fasting, self‐inflicted wounds, exposure to cold, and other mortifications. Crucial to the understanding of this tendency—referred to by Esther Cohen as “filopassionism” (the search for pain in order to imitate Christ) (Cohen 2010)—is the devotion to the human figure of Christ, the idea of the incarnation of God in a living, human body.^13^ Bernardo hugged and kissed the crucified Jesus (who appeared to him), and the Virgin let him suck from her breasts. This devotion to the Incarnated Christ was consolidated by the female German mystics Gertrudis of Hefta, Mechtilde of Hackeborn, and Mechtilde of Magdeburg (Vandermeersch 2014) and, together with the aforementioned “mystic theology,” opened a new space for female “incarnated” religious experiences. Gertrudis felt that God had kissed her in her soul, and she perceived clear details of the incarnated Christ, like his heart, whose beat Mechtilde of Hackeborn also felt (Constable 1982; Carpinello 2006). This derives from the Son of God being incarnated in a mortal body, meaning he was born as a human child and suffered in his human body. Women may not have had access to the theological debates, but they were able to explore their religiosity by feeling tenderness toward a divine baby born in a stable or a strong empathy for his suffering from unjust torture. Moreover, the fact that the Son of God suffered in a mortal body is seen as an invitation to follow him by suffering physically.^14^ Teresa of Avila supports the idea of the incarnation of Christ: In her visions, she sees Christ's body in the form of a living body, not as a mere “abstract” reality, and she experiences her relationship with him not in a purely intellectual way, but through the senses. An example is the paragraph of *Way of Perfection* wherein she describes the physical effects of Christ's sufferings and the expression of goodness in his eyes: “Or behold him burdened with the cross, for they didn't even let him take a breath. He will look at you with those eyes so beautiful and compassionate, filled with tears; he will forget his sorrows so as to console you in yours, merely because you yourselves go to him to be consoled, and you turn your head to look at him” (Teresa of Avila 2012, CV 26, 5). Christ exists in the flesh; he is not immaterial or formless like an idea (V 28, 1).

## Feminine Ascetic Suffering as an Embodied Experience

I propose to study Teresa's experience of suffering by assuming, like Wilhelm Dilthey, that suffering is “lived” and the *Erlebnis* (“lived‐ness”) of suffering (including the fact that it is experienced “for me,” which includes an impression and an image) is the first element of what I call “experience,” the perception of suffering (Dilthey 2013).^15^ Mere perception does not yet entail reflection about the perceived facts, but precedes it. The second element of the experience of suffering is its expression. Expression consists in how individuals and groups manifest their perception, the way in which they convey to others what happens to them—for example, whether they express it artistically, or simply scream, cry, or gesture, as well as other more spontaneous reactions. Expression can modulate perception, as I will explain later. The third element of the experience of suffering is management, that is, how suffering is confronted, avoided, accepted, denied, and so on, as well as the strategies designed to deal with it. Understanding an experience of suffering entails unraveling the conditions of how the experience is situated in the flux of the individual's life and the flux of the individual's life in a community, in a certain place and time.

Teresa's conception of the self includes the strong conviction that the body and mind are two intrinsically connected realities of human beings (V 22, 10), indissolubly united while they are alive, and that one can and must influence one's own body in order to placate mundane passions and to promote spiritual virtues that bring the person closer to God.^16^ That is not to say, however, that the body corresponds entirely with the mundane and the soul with the divine part of the person, as I will explain later.^17^ Spiritual experiences are felt and described in sensory terms; the “celestial bread” can also feed the body (CE 34, 7, 3), like communion, and the spiritual joy when one is “touched” by God reaches and is expressed through the body (thus it is not simply “spiritual”) (CV 31, 2).

Teresa, and other mystic women, did not simply reject the body, though it may seem so at first glance considering their practices of mortification; rather, they were usually able to invent a new body (Romagnoli 2006). The prolonged and frequent fasts that resulted in the cultural phenomenon called “Holy Anorexia” by Rudolph Bell (Bell 1985; Bynum 1987) have been interpreted as a form of madness or mental illness, but this interpretation is not convincing because of the obvious cultural patterns common to the experiences of all these women. This article shares the perspective of Alessandra Romagnoli, who postulates, contrary to other explanations of this phenomenon, that Holy Anorexia and its brutal consequences in the form of deformed bodies, sores, emaciation, and so on, are practicing women's attempts to shape and creatively configure their own bodies (Romagnoli 2006). Refusing to eat may be one of the few ways that women in the Middle Ages and the modern era had to achieve a certain degree of autonomy, given that the main functions attributed to them and their bodies were to be available for perpetual service to male sexual pleasure, pregnancy, delivery, breastfeeding, and servile work. This was not the case for men. Although oppression of men did of course occur, the root of their oppression lay with hierarchical structures rather than with their gender. Except in cases of slavery or total domination, men could generally appropriate their own bodies in several ways, and if they chose the ascetic path, this was not a reaction against a social order that oppressed them as men, as was the case for women under a patriarchal order. There is an impulse of rebellion in female practices of starvation and mortification: an attempt by women to liberate themselves from the social obligations imposed on them as women and to make the choice to surrender themselves in a different way—not to men, but to God. They adored God, incarnated in a male body, and they did it body and soul, believing that it was better to serve divinity than to serve mortal men. Since they were excluded from other languages, they spoke the language of the body, filling and covering it with symbols. Fasting and mortification had a concrete purpose for female medieval mystics, who tried to appropriate their own bodies by rejecting the functions that society assigned to them and transforming them into religious symbols.

Yet one can also argue that mortification was imposed by male ecclesiastical authorities in order to control women. Ascetic practices, obedience, and humility dedicated to surrendering one's will to God (to be like a slave to Him) leave the individual in a vulnerable position. Although being “vulnerable” to God is the aim of this surrender, it poses the risk of other people taking advantage of said vulnerability, manipulating such souls without will, and even promoting unquestioning fealty to male authority figures. Mortification, depending on its context, can be destructive and self‐annihilating, particularly in the case of women, who are traditionally disempowered. In Teresa's case, her use and experience of suffering show the paradoxical affirmation of her personality through her denial of certain parts of herself, her self‐humiliation, and other ascetic practices. However, this was not easy, not least because she had to come to terms with the doubts of her confessors and friends regarding her mystical experiences (as a woman, she was regarded as inherently less trustworthy), a fact that considerably undermined her own self‐trust (V 28).

Teresa used two resources in order to confront two disadvantageous situations of female mystics: lack of theological knowledge and mistrust of feminine religious experiences. For one, she was very conscious of her lack of theological knowledge, but she managed to supply it with imagination, creativity, and a brilliant use of colloquial language, metaphors, and other literary resources in order to describe her own experience. In this way, she created a completely original work rather than an erudite one, as the vast majority of her male coevals did, like John of the Cross, who drew on his immense knowledge of theological texts and the Bible in his writings. For another, Teresa was well aware of the weaker position she was relegated to as a woman, but she resorted to rhetorical humility as a resource to support her testimony^18^ and defend the veracity and power of female faith.^19^ She mentions the “mistica teoloxia” at least once in V 7 in the context of her mystical experiences, with the double aim of distinguishing them from “mere visions” and thus giving them credibility, and emphasizing the opportunity that mystic theology opened for women, mentioning that the Virgin did not need to understand in order to accept (CE 6, 7). In this manner, the path of Christian love was opened for women.

## Teresa of Avila's Perception and Expression of Suffering

For Teresa, suffering is not something intrinsically good or bad, as it depends on where it originates and why one suffers. Living a virtuous life does not guarantee a life free of suffering—on the contrary, virtue is difficult to uphold, full of inconveniences, work, and effort, but it guarantees spiritual rewards. Likewise, living a sinful life does not guarantee pleasure and the avoidance of suffering; it paves the way to Hell, where terrible suffering awaits.

Teresa uses a rich vocabulary to refer to suffering, disturbance, sorrow, and pain: “travajos” (toils), “penas” (sorrows), “sequedad” (aridity), “tormento” (torment), “ansia” (anxiety), “esfuerzos” (efforts), “ímpetus” (impetus), “me revuelve toda” (it disorders me), “me deshago” (I come undone), “llevar la cruz” (to carry the Cross). The worst kinds of suffering she can imagine are described in her conception of Hell: extreme temperature and the feeling of being burned associated with an oven; the disgusting smell and texture of the pestilential mud; unpleasant and poisonous vermin; anxiety, darkness, and suffocation of immurement; fire in the soul; corporeal pain. She perceives and expresses suffering by referring to her cultural and religious symbolic universe and her personal experience as a sickly person. We know how she perceived suffering because she expressed it extensively. Expressing suffering entails a modulation of perception. Little by little, expression becomes something more than mere description (the description is in fact modulated by her symbolic references) of what is happening to her, and slowly becomes true management of suffering.

Teresa distinguishes between physical pain (“dolores y mal corporal,” V 31, 3) as a result of an illness, for example, and spiritual pain (“pena espiritual,” V 30, 1), like the restlessness (“inquietud”) and unrest (“desasosiego”) of the soul. However, as mentioned above, she perceives the body and soul as two intrinsically connected realities of the human being (V 22, 10). Teresa constantly refers to sensorial elements when she describes spiritual experiences, and the body always plays a role in them. For her, it is possible to feel corporeal pain without spiritual pain (and this is always more bearable), but sometimes they are linked, which makes it more difficult to bear them (V 30, 8). The relevant distinction here is not body/soul, but divine/mundane—in other words, whether something serves to achieve perfection, the impulse of love destined to deserve God's love, or serves mundane interests.

Suffering is desirable (including physical pain), except when it comes from attachment to the world, because this hinders the relationship with God. However, physical pleasure is not desirable, and must be renounced on the way to perfection (CV 12, 3).^20^ Thus renouncing the world means to renounce the pleasures of the body, but not to renounce the pains of the body. What is good for the soul may be bad for the senses (painful), but the suffering of the senses may be good for the soul. There are three acceptable kinds of joy, if they come (1) from good things that happen to us in the world, (2) from virtuous actions (M 4, 1, 4) or from thinking about the glory that awaits virtuous Christians and the love of God (V 12, 1), or (3) directly from God (the “gustos,” or “tastes” of God). The first two kinds of joy are felt by the senses, and they are passions of the soul (meaning that the body/soul dichotomy does not apply).^21^ The origins of joy subsumed in (1) are not bad, but those encompassed in (2) are better because they are connected to God (even if they begin in our nature, they end in God). The joys described in (3) are entirely different because they do not come from our nature, but from God. Only the “tastes” of God widen the heart (M 4, 1, 4). Renouncing the mundane pleasures (honor, wealth, physical pleasures, and so on) always comes with great delight and spiritual joy (V 13, 3). The joy that comes from God is incomparable to mundane joy, “all of which are but dung” (Teresa of Jesus 1946, 174, V 27, 12).

When Teresa describes her feelings during the ecstasies she has experienced, she, similarly to other mystics, always uses contradictory terms. What she feels in these circumstances is not pure joy, but a mixture of emotions. God sends an arrow or a spark into her soul, and such a spark “excites” or “ignites” the soul—though it is a pleasant fire. It burns but does not burn *her*, and it is delightful (“deleitoso”). Only God is able to join pain with the quietude and the taste (“gusto”) of the soul. She describes this state as a “delightful inflammation” (literally, an “inflamación deleitosa” [M 6, 3, 8]).^22^ We find a similar description in *The Book of Her Life,* in which she relays her vision of an angel:

In his hands I saw a long golden spear and at the end of the iron tip I seemed to see a point of fire. With this he seemed to pierce my heart several times so that it penetrated to my entrails. When he drew it out, I thought he was drawing them out with it and he left me completely afire with a great love for God. The pain was so sharp that it made me utter several moans; and so excessive was the sweetness caused me by this intense pain that one can never wish to lose it, nor will one's soul be content with anything less than God. It is not bodily pain, but spiritual, though the body has a share in it indeed, a great share. So sweet are the colloquies of love which pass between the soul and God…. (Teresa of Jesus 1946, 192–93, V 29, 13)

The symbolism of the fiery dart piercing her heart and taking a part of her internal organs with it is powerful. She feels an extreme pain, which she considers at the beginning to be wholly spiritual, but finally acknowledges its corporeal nature too. Although it is an extreme pain, it is “soft” and she does not want it to stop.

During the ecstasies, the senses of the body are partially or totally suspended, the hands and body are cold as if the body were bereft of its soul. The body returns briefly to life before slipping into the state of ecstasy once more. The retirement of the body entails giving more life to the soul (M 6, 4, 13). This is exactly what she expects, what only God can give her: to suspend the mundane elements of herself (the senses, the exterior human being) in order to increase the divine elements of herself (her soul, her interior life). The ascetic method has such an objective. However, complete separation from the body is not possible, and her mystical experiences are corporeal, as they all have consequences for her body and manifest through the senses (pain, joy, and so on), yet they also confuse the senses because they are sent by God, whose nature is wholly different from the ordinary, mundane things the senses are used to perceiving. During the ecstasies, the senses and potencies are suspended, as is reason. However, Teresa can still feel; her ecstasies are not states of blankness, but intensely emotional spiritual experiences. The mystic experiences transform Teresa's body, psyche, and biography deeply.

## Teresa's Sufferings

It is possible to list the causes of Teresa's suffering:

1. Various illnesses cause her pain, inconvenience, disturbance, and sorrow (V 4, 5–6; V 5, 7; V 7, 11). Medical treatments are painful and distressing too.
2. Mundane issues and her attachment to the world in itself—”trials, persecutions, back‐bitings, and infirmities” (Teresa of Jesus 1946, 112, V 19, 3).
3. Conflicts between her desires and her capabilities: (a) Having a weak body makes her feel “confined” because her body does not allow her to serve God as she would like to. For example, she cannot make penitence as she would like. Eating and sleeping are painful for her, but she must not stop doing them, so she eats and sleeps as an obligation to continue living in this moral way (CC 1, aa). (b) Her impetuous desire to love and serve God makes her feel that she is able to face any kind of suffering, but finally she realizes she cannot. This conflict between what she wants and what she can do is a source of strong suffering (she feels that she “comes undone” (“se deshace entre sí”), as if she were split into her desires and her capabilities [CC 1, a4]).
4. Not only having a body and having to bear temptations (V 31, 3), but simply having to live a mundane life in which it is impossible not to sin. She desires to follow only God's will (E 6), but expects to lose her resolve to do so and thus to sin. The desire to be with God entails suffering too—however, God is merciful and alleviates it with the joy of the union (M 6, 4, 3).
5. Penitence. Teresa suffers when she remembers her past sins (V 1–4). During one period in her life, she does not even dare pray because of the sorrow caused by the idea that she has offended God with her sins (V 6, 4). The pain of one's own sins grows the closer one is to God (M 6, 7, 1).
6. The sins of people who do not appreciate divine love and who offend God (E 3, 1). She suffers because she knows of the torments they will suffer in Purgatory and Hell (E 10, 4 and E 11, 1).
7. Humility. She suffers when receiving the grace of God because she considers herself to be undeserving and unable to do enough to show her gratitude. Not knowing if her humility is authentic or a trick of the devil also causes her pain (V 31, 014).
8. Empathy with the suffering of Christ (V 8, 6). Upon waking up, she sees a picture of Christ with sores, and empathizes so deeply with his pain that it causes her to suffer in turn (V 9, 1). She feels able (but unworthy) to comfort him. From the moral point of view, empathizing with the passion of Christ is considered a worthy pain (“pena meritoria”). There is a virtuous joy (“gozo virtuoso”) too, in thinking on the promise of Heaven and the love God has for us (V 12, 1).
  God causes deliberate suffering to humans in order to test them, to see if they will be able to “drink the chalice and help him to carry on the Cross” (V 11, 11). However, this always comes with a reward, even before death (V 11, 11). Suffering is a sign of divine love: if you suffer more, God loves you more (CE 32, 7, 1). The love of God is shown in works (“travajos”), “rough death” (“muerte áspera”), torments, suffering everyday injustices and forgiving those who caused them (CE 3, 11), and extreme illness (M 6, 1, 7).
  Teresa perceives Jesus’ suffering during her prayer of contemplation not merely as a historical fact that belongs in the past, but as if Jesus were permanently and continuously suffering for all human beings. Jesus suffered loneliness (when he went to the orchard), torture (“bound to the column, filled with pain, with all His flesh torn in pieces for the great love He bears you” [Teresa of Avila 2012, CE 26, 4–5]), prosecution, contempt, and humiliation; his friends denied him, he felt cold and the burden of the Cross (CE 26, 4–5).

- The union with God causes a kind of pain, a “delicious pain” that is better than any mundane joy: “the soul would gladly be dying of this ill” (Teresa of Jesus 1946, 191, V 29, 10). She describes the transverberation as a “soft pain,” a great pain (“pena”) joined with a great pleasure (“gusto”). This is a “spiritual pain,” that is, something that occurs between the soul and God, though the body plays a role as well.
- The spiritual search for God entails many disturbances and difficulties (“sequedades”), particularly at the beginning. It is important to persevere and to stay in control of one's senses. The contemplative life of Teresa's nuns includes great suffering and sacrifices: “Their duty is to suffer as Christ did, to hold high the cross, not to let it out of their hands whatever the dangers they see…” (Teresa of Avila 2012, CV 18, 5, 3).
- Mortification. Teresa mortifies herself. She says to her nuns that penance is not wrong if it is done in a way “so as not to harm their health” (Teresa of Avila 2012, M 3, 2, 7). Self‐inflicted pain is not included in the monastic rule but is also permissible in order to achieve perfection. The mortification should be adapted to a person's individual capacities. Obedience to the rule and one's superiors prevails over the individual desire to make penitence (VD 18, 8).

## Teresa's Management of Suffering

Teresa is able to use detachment from the world as a resource for managing illness. Her attitude toward illness changes from the beginning of her spiritual development. She considers illness and health to belong to the set of mundane issues from which one needs to liberate oneself: “As my own health is so bad, I was always impeded by my fears, and my devotion was of no value at all until I resolved not to worry any more about my body or my health; and now I trouble about them very little…. Since I have been less self‐regarding and indulgent my health has been very much better” (Teresa of Jesus 1946, 76).^23^ At the beginning of her spiritual development, Teresa is still too concerned with mundane matters, but little by little, she frees herself from them. At this point, suffering because of her own health is seen as a lack of love for God because it is a consequence of not giving oneself totally to Him, so Teresa fights against this kind of sorrow. In parallel, she feels that her body is like a prison that prevents her from being with God and she desires to die. However, after her spiritual marriage to God (in which the senses are suspended, and the flesh seems to be diminished to give more room to the soul, although the person is still “incorporated”), she no longer desires to die and becomes comfortable with her body because she assumes that being alive and having a body entails ways of serving God that are impossible in the other life (M 7, 3, 12). Spiritual marriage gives her a kind of peace that can be understood as self‐acceptance.

Moreover, liberation from mundane issues entails a different attitude toward illness, which results in her feeling healthier. The perception and the symbolism change: Illness decreases in importance, so she does not feel as ill as before. Not caring too much about the health of the body is part of retirement (“desasimiento”) from mundane life, business, worries, honors, and social recognition, which includes retirement from one's own will too.^24^

She admonishes the nuns of her reformed monasteries not to complain about their pains and to become used to not satisfying the body (CV 11, 2) and, what is more, they must be ready to die for Christ (C 2, 10, 5, 3). Renouncing one's own care is the next step to renouncing one's pleasure. Renouncing one's own life is the next step after the renunciation of one's own will. In such renunciation Teresa finds great joy (CV 12, 3), even if this may seem contradictory. The contradiction disappears if we think that renouncing mundane pleasure and self‐care comes with spiritual joy, with divine reward, which is much better than either. Being able not to care about death, health, and the body in general is a consequence of renouncing one's own will and leaving oneself in the hands of God; it is part of the spiritual process. Simultaneously, this entails a particular kind of control over one's own body, a particular attitude consisting in not succumbing to its demands.

Another management of illness consists in interpreting it as a divine gift (CV 10, 6, 4). This applies to the troubles, work, and aridity (“sequedad”) that one feels when one sets out to follow the spiritual path (“I believe myself that often in the early stages, and again later, it is the Lord's will to give us these tortures, and many other temptations which present themselves, in order to test His lovers and discover if they can drink of the chalice and help Him to bear the Cross before He trusts them with His great treasures” (Teresa of Jesus 1946, 67). God makes human beings suffer, which can be interpreted as a divine gift in the sense of a proof of engagement of believers with divinity, and a test of the strength of one's commitment. Illness can be interpreted as divine punishment for not making penitence (V 24, 2) too, in the sense that such illness substitutes for penitence. After all, nobody is completely innocent. This interpretation is consistent with the economy of the salvation.

Teresa asks God to make her suffer (“I desire to suffer, Lord, because Thou didst suffer” [Teresa of Jesus 1946, 68, V 11, 12])^25^albeit not in a masochistic sense, since she does not derive any pleasure from the suffering she desires. Instead, she desires to suffer (*filopassionism*) in order to imitate Christ (a wife of Christ should be willing to share with him his disgraces and toils [“deshonras y trabajos” CV 13, 2]), not only his glory (CE 26, 6 too); to suffer *for* God (as a service to God) as a consequence of loving him; as penitence for one's own sins (CV 13, 1); and as penitence for the sins of others (for example, to liberate souls from Purgatory). Christ suffered, and he did not deserve it. Mortals, however, deserve it, hence there are far more reasons for them to suffer, and they should therefore embrace it. In this sense, mortification is accepted as part of the ascetic way of life, and it is acceptable to deliberately search for suffering. However, life must be preserved and cared for. Excessive penitence is not tolerable, and sick nuns are cared for and supported in the monasteries (Cs 7, 3). Although one should not care too much about one's health, there is an obligation to live and serve God here in this world. Teresa finally assumes that it is possible to serve Christ in this mundane life in a way she cannot do afterwards (CC 46), and this provides a particular value to mundane life. This obligation includes caring about the health of others, as compassion and care are dimensions of love. Anyway, nobody should mortify themselves to the point of risking their lives.

At the beginning of her spiritual life, Teresa desires suffering as a way to love and serve God, but is at the same time afraid of not being able to withstand it. By asking God for the strength and grace necessary to persevere through this proof of her love, Teresa manages the conflict between her desires and her capabilities (CC 1, 24; V 12, 17). She concludes that God does not send more suffering than what one is able to bear, and this is liberating to her: she becomes sure that she does not have to bear more suffering than she is able to stand. The strength she feels that God provides her is also a resource for the management of suffering. Both suffering and the capacity to endure it are seen as signs of divine love. God sends more suffering to those who love more, and the great suffering of Christ is proof of it. Teresa argues that if God decides not to relieve pain or suffering, this is the best for the soul (F 28, 18).

If it comes from God or is dedicated to Him, suffering is something positive. In this case, one should be able to appreciate and be grateful for it. When the pain is unbearable, Teresa asks God for patience and to prolong it until the end of the world:

When the pains and the bodily suffering are quite intolerable, my custom is to make interior acts as well as I can, and to beseech the Lord, if it be His Majesty's good pleasure, to give me patience; if only I have that, I can keep on suffering in this way until the very end of the world. So, when on this occasion I found myself suffering so severely, I took to these acts and resolutions, using them as means which would enable me to bear the pain. (Teresa of Jesus 1946, 204–05; V 31, 3).

This determination, which she considers to be supported by God, helps her to endure by “manipulating” the pain (in principle it is undomesticated, wild) by desiring both the pain and its endurance as acceptance of the divine will. In this way, she accepts the pain on her own terms so it does not overwhelm her.

The Catholic ritual of Communion is also considered to be a resource to manage suffering and pain, associated with a direct improvement of one's physical health and the strengthening of the soul in the case of illness. Communion represents not only spiritual consolation, buta physical relief (CC 1, a). Its symbolic dimension represents the reparation of the body/soul. This can be interpreted as a case of “symbolic efficacy.” An example of this kind of efficacy is offered by Claude Lévi‐Strauss in his description of a shamanic cure among the Kuna People in Panama, and reinterpreted by David Le Breton from a nondualistic perspective of the person. According to Le Breton's reinterpretation, it is not the case that the ritual has a psychological impact and therefore an effect on the body (and is thus able to cure the physical condition), since this explanation is still based on the premise of mind/body dualism. In Le Breton's view, the symbolic dimension of the ritual reintroduces the meaning of the stressful situation by reintegrating it in a symbolic universe of meaning (the myth), and provides support because it reaffirms the position of the sufferer as a member of the community that shares the same symbolic universe. Mind/body dualism does not persist in this explanation because the body is conceived as intrinsically symbolic. In short, symbols are so effective because bodies themselves are symbolic (Le Breton 1999).

Teresa's symbolic universe includes an economy of salvation. She assumes that there is a correlation between sins and the penalties for such sins. All human beings are guilty, even if they are not aware of their sins. One needs to pay for one's own sins, and it is better to do so in this world, because the punishments of Purgatory or Hell are much worse. Suffering plays an important role in this economy of salvation. The ascetic exaltation of humility includes the idea that one must maximize the perception of one's own sins in order to pay enough for them.^26^ In doing so, one expects a reward, and this reward is guaranteed after death in the form of the salvation of the soul, the vision of God, and/or Paradise, but it can be received to some degree even in mundane life (V 11, 11), although this may take some time (CV 17, 2). Everybody can achieve perfection in order to become deserving of the salvation of the soul and eternal life, both by following the way of the active life or the way of the contemplative life and by doing penance for one's own sins. In this calculation, the end, or reward, is salvation. This seems to contradict the definition of love for God, because if one loves God, one should be able to accept His will even at the risk of not achieving the reward. But according to Catholicism, God's will is not arbitrary. There are certain rules, like the promise of salvation, as well the threat of Purgatory and Hell. More suffering during one's life in this world entails less suffering in Purgatory (V 38, 29). This does not present a contradiction, because salvation consists of divine love. However, there is still a difference between conducting oneself according to the economy of salvation, which is a “good inspiration” for Teresa (she recognizes that this attitude moved her in the beginning), and the perfect incentive: not to expect any reward, but simply loving God.

For Teresa, there is a superior spiritual state beyond calculation, namely contemplation. Contemplation is a state of beatitude resulting from God's extreme generosity—no human being can possibly be deserving of it (CV 25, 2), although one can work to be ready for it, so to speak, through ascetic practice. In such a state of beatitude, love transcends any calculation. It is a kind of suspension, a rest in God, the complete surrender of the individual will and freedom: “He [the soul] feels so happy merely with being close to the fountain that he is satisfied even without drinking. It doesn't seem there is anything else for him to desire” (Teresa of Avila 2012, CE 31, 2–3). Love means union for Teresa.^27^ Perfect contemplation entails falling in love with God and joining one's own will to God's will. In the state of beatitude, calculation has no purpose because intellectual or fear‐driven considerations cease to matter. The believer surrenders to God and God receives her.

Two attitudes coexist in Teresa: the calculation of blame and punishment, thus understanding unavoidable suffering as a penitence for one's own sins (or for the sins of others), as well as the tendency to love God without any further interest. Not expecting to deserve any reward from God is a good way to obtain a reward from him (M 2, 10). Choosing suffering to imitate Christ is a sure way, even if there are other ways to find God. Teresa declares she would choose suffering in order to imitate Jesus Christ even if there were no other benefit (M 6, 1).

The different resources that Teresa used to manage suffering are: (a) detachment from the world, (b) understanding it as a step to spiritual enhancement that comes with a reward, (c) the economy of salvation, (d) love for God (imitation and understanding suffering as a service or a proof of love to him), (e) receiving from God the strength to endure, and (f) ritual communion. Such resources usually entail palliations of initial suffering, consolation, or at least the placement of them in a symbolic universe of meaning, which is crucial to bearing them.

## Conclusion

Teresa reformed the Carmelite order with the aim of designing a demanding way of life, dedicated to facilitating ascetic practices and the spiritual development of those women able to undertake the challenge. This spiritual development and its associated practices extended beyond mere austerity to include mortification, self‐humiliation, and obedience. At the same time, Teresa was aware of the fact that she was opening valuable spaces of female creative cooperation and self‐development in which women were liberated from the social burden of servility to men, and that she had an opportunity to further their intellectual development: Since the nuns in the monasteries were liberated from the confines of marriage, sexual obligations, the burden of continuous pregnancies, deliveries, and breastfeeding periods, as well as servitude to men, they were able to devote themselves to intellectual pursuits. Although Teresa at times found it difficult to serve God, she nevertheless considered it far preferable to serving men, and she was able to interpret the self‐confidence, maturity, and intellectual position she achieved as divine inspirations, which reinforced them. Framing certain kinds of suffering as positive was part of this process. On the one hand, this was proof of the veracity of her message and experiences: Her willingness to suffer and die like a martyr was intended to demonstrate that she was not an impostor. On the other hand, she transformed the suffering that she initially simply endured into a productive force to drive her spiritual development. By desiring suffering in order to imitate or to serve God, by understanding it and her capacity to embrace it as proof of divine love, and/or by interpreting it as a part of her atonement for her own and others’ sins (which might spare her and others worse suffering in Purgatory or Hell), she actively managed her suffering, transforming it into something she was able to control instead of passively endure. At the same time, she challenged the idea of the fragility of women, showing that women could be much more than what was generally attributed to them.

Of course, this feminist interpretation of Teresa of Avila stands in direct opposition to that of the Spanish Catholic tradition. More research is also needed in order to evaluate to what degree Teresa's influence on female education during the ensuing centuries has contributed to a different model of woman as one who is available to suffer for others but not exactly able to transform her capacity to confront suffering in order to turn it into a source of self‐confidence and strength.

Teresa's management of suffering was effective because she assumed that the whole self is manageable by internal forces (like one's own will or passions) and mundane, divine, or demonic external forces, but nevertheless something one can learn to dominate. At the same time, the body is not merely the recipient of mystic experiences, but a necessary element for their expression and “performance,” as it has a symbolic character. The idea of the Incarnation of Christ is crucial to understanding this belief, together with the concept of *filopassionism*: It is much easier to empathize with Christ's sufferings if it is assumed that he suffered them in a mortal body. The idea that the Son of God suffered by bearing sorrow, pain, and humiliation can be extremely comforting for those in similar circumstances. Those whose lives are mired in toil and trouble, and especially those who live at the margins of society, see their suffering recognized and even elevated in importance. However, two main problems can arise from this understanding of suffering.

The first is that the comforting dimension of the Christian ascetic‐mystic view of suffering depends to a large degree on its religious symbolic universe. Nevertheless, if we assume a postmetaphysical perspective, then we need to look for other symbols and other sources of meaning for our suffering, which is not an easy task because these alternative resources can hardly be as effective a provider of meaning as religion. Even though it may be considered fictive from a postmetaphysical perspective, religious dogma can give an internally coherent and complete explanation of everything, something that science cannot do. Secular theodicies can reproduce the same problems we can find in religious explanations. As Veena Das points out, states or even social movements can create communities of suffering that homogenize or minimize individual experiences of suffering (Das 1997), which is a risk of any collective management of suffering. In the political realm, Teresa's experience may be of relevance inasmuch as she manages suffering actively, as part of her own circumstances, instead of simply passively accepting it as it came, and although she managed it in the symbolic universe of Catholic dogma, she did it in a way in which her individual experience was not denied or minimized, but acknowledged. Yet it must be considered that transforming individual suffering into collective action does not automatically entail this action being oriented toward freedom and the emancipation of the oppressed, or that the suffering of individuals is recognized as such.

The second problem is that the Christian ascetic‐mystic view goes one step beyond managing unavoidable suffering; even if there is an obligation to care about the suffering of others and limits to mortification, suffering has a positive dimension in the spiritual development of a believer. This may be a reason to seek out suffering deliberately, as in the case of mortification and self‐humiliation and even to justify existing (avoidable) suffering as beneficial to the one who suffers.^28^ This kind of justification for suffering can become a reason for not fighting against it; for example, it could normalize social injustice.^29^ Framing suffering in a negative way, however, provides considerable advantages, since it is possible to discover new ways of managing it and to mobilize more resources for its management. Furthermore, the negative view of suffering does not deny that it can be an opportunity for developing one's strengths, for converting them into artistic or intellectual work that can help the creator (in a kind of sublimation) as well as others, or for developing other capacities. All these resources are positive in the sense that they confront suffering, which persists as something to *be* confronted. The fact that human beings are capable of making a virtue of necessity does not entail that necessity *is* a virtue. There is an argument to be made that a particular kind or particular level of suffering may be justified, necessary, or even desirable in certain contexts if it serves to avoid worse suffering in the future, yet there is little to be gained from simply declaring suffering *per se* a “morally good” thing without at least making an attempt to eliminate its causes whenever possible (for instance, in cases of social suffering caused by social injustice or institutions), and to support, help, or comfort the suffering regardless. Of course, this was sometimes Teresa's calculation too: Mortification was seen as a way of avoiding suffering in the life beyond death or helping the suffering souls in Purgatory. Yet this particular calculation depends on Catholic dogma.

Independent of her positive conceptualization of suffering, Teresa's management of it is still interesting inasmuch as she is able to actively manipulate and control it. Her attitude of accepting unavoidable suffering to the point of desiring it is associated with renouncing her own will, that is, the acceptance of one's suffering as the will of God. Nevertheless, the opposite interpretation is also possible: In accepting their suffering, people can affirm themselves; by desiring suffering one cannot avoid, one can master it. The subjugation of suffering to one's own will, the assumption that suffering belongs to oneself or one's own circumstances and that one can use it as a catalyst for something positive (for example, to become stronger, to help others in similar situations) can increase one's strength to bear it. Like the contrary perspective (rejecting suffering as something that is not part of oneself in order to fight against it), this is a very valuable management of suffering.

There is one more risk in the Christian ascetic‐mystic perspective on suffering. The monastic way of life can be seen as a model of pacifistic common life. Giorgio Agamben sees Franciscanism as a model for organizing common life without repression and without exceptions due to its capacity to integrate life, giving meaning to life, the guarantee of care, and the possibilities for spiritual development (Agamben 2014). However, this model becomes integrated into a greater hierarchical power structure that leaves ascetics in a vulnerable position, susceptible to manipulation by authority figures for their own gains and purposes. Moreover, especially in the Catholic context, these authority figures are always male, which can easily add a gendered bias to the detriment of the obedient women. Pastoral power can be comforting in the sense that one finds spiritual support and one's free will is suspended, so to speak, as others make the relevant decisions, but it represents an incursion into the most intimate parts of a person. It strips the soul bare, thus leaving the person at the mercy of possible manipulation and exploitation. Although Teresa herself did accept the overall authority of the Catholic Church, she questioned the pastoral power of every spiritual director and always looked for the best counselors of her time, contrasting their opinions and recommendations about her interior life and her decisions. Finally, she followed what she considered to be God's expectations of her. The stories of the great mystics we know are the stories of very strong women and men, able to paradoxically affirm their personalities through denial of certain parts of themselves, self‐humiliation, ascetic practices including mortification, and renunciation of mundane issues. However, it is difficult to know the stories of those unable to endure this process: those who were annihilated by it.

## Notes

1 See, for example, David Pearce's proposal to abolish suffering, “The Hedonistic Imperative” (Pearce 2007).

2 This assumes José Ortega y Gasset's idea that we “are” in our beliefs, meaning that we assume them without further reflection as part of the resources we become to manage life and world (Ortega y Gasset 2005).

3 Other, similar collective performances can be found elsewhere in contemporary Europe. Two crucial, more recent examples of similar ascetic‐mystic perspectives on the experience of suffering are Gemma Galgani (Camigliano, Italy, nineteenth century) and Marthe Robin (Châteauneuf‐de‐Galaure, France, twentieth century). See Galgani 1997; Robin 2012.

4 See, for example, the expressions used to apologize in different European languages (all of them can be translated as “sorry”: “lo siento“ in Spanish (literally “I feel it“), “desolé” in French (literally “disconsolate,” “desolate”), “Entschuldigung” in German (“ent‐” meaning “the removal of something,” and “Schuld” meaning “guilt,” so it literally means “removal of guilt,” implying the speaker's desire to bring his feelings of guilt out into the open and be rid of them) or even more, “Tut mir Leid” (literally “it makes me suffer”). “Sorry” has the same root as “sorrow.” Apologizing is thus not simply acknowledging a bad action, but feeling bad as a consequence of it, like contrition in the sacrament of penitence.

5 See a list of recent studies showing how suffering is still seen as a possibility for redemption after a transgression and as a resource of self‐indulgence in Leknes and Bastian 2014. However, the authors neither mention the cultural background of the cited studies nor where they were developed. Their argument consists in looking for the “benefits of pain”; the different sociological studies are mentioned as part of a “universal” perspective on suffering.

6 For example, Angela of Foligno (thirteenth century) did not become a nun (Brezzi 2006); Dorothea of Montau was married, had nine children, and became an anchorite after the death of her husband and children in the fourteenth century (Wallace 2011); Margery Kempe was a “contemplative pilgrim,” married, and a mother of fourteen children (Dickens 2009); Mary Ward of Yorkshire defied papal authority in the seventeenth century when she defended the right of religious women to form an apostolate outside of the convents (Wallace 2011).

7 As explained by Olegario González de Cardedal, Pseudo‐Dionysus represents a crucial moment in the use of the term “mystics” in the Christian context, because in the *Mystic Theology* he describes the “experimental” knowledge of God that goes beyond intellectual knowledge. It was Hugh of Balma who clearly distinguished in the thirteenth century between “mystics” and “scholasticism” in his work *Theologia mystica* (prol. 7), and the idea that love, as well as intellectual knowledge, was a way to know God: “sola amoris unitive regula ad ipsum qui est fons totius bonitatis, Spiritum dirigente. Haec igitur est mystica theologica, id est occultus sermo divinus, quo mens ardore amoris disposita, linguis afectionum Christum suum dilectum occulte adloquitur” (cited by González de Cardedal 2015). See the English version: “steering the spirit solely by the rule of unitive love toward him who is the source of all goodness. This, therefore, is mystical theology, that is, the hidden divine word with which the human spirit, disposed by ardor of love, converses secretly with Christ, her beloved in the language of the affections” (Balma and Ponte 1997, 71). Hugh of Balma's work was translated into Spanish in 1514, and it influenced Francisco de Osuna's work *Abecedario espiritual*, a book that had a strong impact on Teresa of Avila, as she recognizes in *The Book of Her Life* (V 4, 7; see note 9 for explanation of reference numbering).

8 Teresa declared that she was not troubled by the menace of the Inquisition because “for the Church or for any truth of Holy Scripture I would undertake to die a thousand deaths” (Teresa of Jesus 1946, V 33, 5).

9 All the references to Teresa's works in Spanish follow Santa Teresa de Jesús 2006. In English, I follow Teresa of Jesus 1946 and Teresa of Avila 2012. I follow the standard conventions of referring to Teresa's works by the initial of the work, chapter, and section numbers. The following abbreviations are used: CC: *Cuentas de conciencia [Accounts of Conscience]*; CE: *Camino de perfección*, códice de El Escorial [*Way of Perfection*, El Escorial codex]; CV: *Camino de perfección*, códice de Valladolid [*Way of Perfection*, Valladolid codex]; Cs: *Constituciones [Constitutions]*; F: *Fundaciones [Foundations]*; M: *Moradas del castillo interior [The Mansions or the Interior Castle]*; MC: *Meditaciones sobre los Cantares [Meditations on the Song of the Songs]*; V: *Vida [The Book of Her Life]*; VD: *Visita de Descalzas [Visitation of Discalced Nuns]*.

10 A thorough discussion about the definitions of “asceticism” can be found in Clark 1999.

11 However, grounding a renunciatory program in the Bible entailed an exegetical problem for the early Christian ascetic writers “because the Bible only sporadically supported their agenda; many verses appeared rather to assume that marriage and reproduction were the norm for godly living” (Clark 1999, 3).

12 Simenon and Daniel lived more than thirty years on the top of a pillar, according to tradition (Palmer 2014).

13 During the first ten centuries of Christianity, the idea of the “Incarnated Verb” predominated, that is, the manifestation of an invisible God in the man Jesus, which is a more intellectualistic tendency inherited from Neoplatonism (initially propagated by Pseudo‐Dionysus) (Vandermeersch 2014). This is the idea of incarnation developed by Augustine of Hippo in *De Trinitate*: The Son, sent by god, incarnates, comes to the world in human form and participates in the history of humanity by transmitting the word of God—hence, the Son is the Verb (Hipona 1968). Francis of Assisi was the main disseminator of the devotion to the humanity of Christ among the people. According to tradition, he was the first to receive stigmata, as well.

14 In this period begins the devotion to the moments of the Passion, culminating in the oration of the Via Crucis (standardized in the fourteenth century), the devotion to the relics of Christ's body like the Holy Blood, the celebration of the Corpus Christi, and the imagery of the Holy Heart.

15 I assume that both suffering and pain are unpleasant experiences that affect the whole psycho‐physical individual. “Suffering” is a more general term. “Pain” is “a process resulting from a somatosensory perception, subsequently present in the brain as a mental image and followed by an unpleasant emotion as well as changes in the body,” whose perception is influenced by cultural, educational, and personal factors. Pain is one of the multiple possible sources of suffering, but it is not the only one (Bueno‐Gómez 2017).

16 In this sense, Teresa assumes Augustine's distinction between the “outer human being” that perceives the exterior world and can act upon it, and the “inner human being,” the part of the person closer to God (Hipona 1968).

17 Teresa adopts Paul's doctrine, according to which the body can be used to sin but it can be used to serve God too (Romans 6:13). The use of the concepts “sarx” (flesh) and “soma” (body) in Christian theology is not clear and consistent. There is a general tendency to associate “sarx” (“carne” in Spanish) with the muscles, nerves, and material corporeity of human beings in general (see, for example, Roldán 2010 on the distinction between flesh and body), although even in Paul's texts the terms “sarx” and “soma” overlap. “Soma” (“cuerpo” in Spanish) usually refers to the physical body, but sometimes “soma” denotes the whole human self (Scobie 2003, 678), the living body in the world or the spiritual body after the resurrection. Teresa uses the word “body” (“cuerpo”) much more frequently than the word “flesh” (“carne”), although she uses “carne,” for example, in reference to the physical wounds inflicted on Jesus during the tortures, in the sense that his flesh was damaged (V 26, 5). She defines the body once as the “outer human being” (“el hombre exterior”) (CV 31, 3), see note 16.

18 In V 15, 8, she states that it is much worthier to study and practice humility than “all the science in the world” (a similar idea is in MC 6, 7). This emphasizes her position as an uneducated woman; humility was something within her reach. Nevertheless, she was not as uneducated as she pretended to be, since she could read and write in Spanish, and she knew the translated works of Augustine, Francis of Osuna, Gregorius Magnus or Saint Jerome, as well as the Bible through liturgy. See Bueno‐Gómez 2018 on the uses of humility by Teresa of Avila.

19 In CE 4, 1 Teresa affirms that God has promoted women's piety, and she says explicitly that he has found “so much love and more faith” in women than in men. Moreover, she refers to the difference between the goodness and justice of God and the justice of the world's judges, all of them men, who find every female virtue suspicious.

20 In V 7, 17 Teresa affirms that the spiritual life is in opposition to “contentment, pleasures, and sensual pastimes” (my translation of “contentos, placers y pasatiempos sensuales”).

21 Teresa associates the passions with perturbations of the soul that can dominate the subject but can be mastered. The concept of “emotion” did not exist in sixteenth‐century Spain, as explained by Elena Carrera (Carrera 2007).

22 Kavanaugh and Rodríguez (in Teresa of Avila 2012) translate “inflammation” as “enkindling.” The original terms used by Teresa are contradictory: “deliciosa” means “delightful,” whereas an “inflamación” (“inflammation”) is usually associated with redness and pain, with damage to the body, that is, something not especially delightful.

23 “Como soy tan enferma, hasta que me determine en no hacer caso del cuerpo ni de la salud, siempre estuve atada, sin valer nada … después que no estoy tan mirada y regalada, tengo mucha más salud” (V 13, 7). The translation is not completely loyal to the original text: she says that she was the one “of no value at all,” not her devotion.

24 Recollection (“recogimiento”), a method of prayer, is also a way of retirement. It consists in withdrawing from the senses and external things (C 99).

25 “Padecer quiero, Señor, pues vos padecisteis” (V 11, 12).

26 On the use of ascetic humility and rhetorical humility by Teresa of Avila, see Slade 1986; Weber 1990.

27 As in the case of Augustine, who defines love as “life that joins” (Hipona 1968, 435). He understands that loving one's neighbors “in God” means to join God in the very activity of loving (assuming that God is love) (Miles 2002).

28 Emmanuel Levinas uses this argument to defend the uselessness and senselessness of suffering (to justify the suffering of others would be the origin of any immorality), and advocates considering it a bad thing. For him, the only meaningful suffering is the generous suffering for others (Levinas 1993).

29 This may be a consequence of Teresa's idea that it is desirable to be slaves to God, and that in response, God may enslave them in the world, as he was a slave too, and this would not be a grievance, but a mercy (M 7, 4, 11). We should abolish all forms of slavery and oppression, because the suffering they cause to human beings is simply unjustifiable, particularly inasmuch as they impede the free development of individuals, and should be seen as separate from whatever value one may assign to suffering for the spiritual development of individuals.
